# The Beneficent Work of the Surgeon

**Published:** 1907-08

**Authors:** 


### THE BENEFICIENT WORK OF THE SURGEON.

S. C. Carson emphasizes the value of surgery. when it is called for, and contradicts the allegation that surgeons are too fond of operating, as not justified by the facts. He does not believe that surgery has reached its limit of progress even in the simple matter of manipulative skill, and thinks that the advances in surgical diagnosis are each year tending more and more toward making simple exploratory incision a reproach to the surgeon who has to avail himself of it. He gives illustrations of the close relation of pathologic knowledge to successful surgery and of the impossibility of routine rules and methods applicable to all cases. The skill and judgment of the operator are more and more to the front, but in the final analysis, after all, the chief elements in good surgery are simplicity and good common sense.—Jour. A. M. A. April 13, 1907.

